# The Impact of microRNA Regulation on Immune Recovery in HIV-1-Infected Patients Treated during Acute Infection: A Pilot Study

**DOI:** 10.1155/2020/5782927

**Published:** 2020-12-03

**Authors:** Yuping Fu, Yan Liu, Zhiying Liu, Lifeng Liu, Lin Yuan, Xizhao An, Jufeng Sun, Tong Zhang, Hao Wu, Shi Lian, Bin Su

**Affiliations:** ^1^Department of Dermatology, Xuanwu Hospital, Capital Medical University, Beijing 100053, China; ^2^The First Affiliated Hospital of Jinzhou Medical University, Jinzhou 121001, China; ^3^Center for Infectious Diseases, Beijing Youan Hospital, Capital Medical University, Beijing 100069, China; ^4^Beijing Key Laboratory for HIV/AIDS Research, Beijing 100069, China

## Abstract

microRNAs (miRNAs) are small noncoding RNAs involved in a large range of cellular activities and can be used as biomarkers and indicators for diagnosis. We investigated the alterations in miRNA profiles in immune reconstituted vs. nonimmune reconstituted HIV-1-infected individuals to assess the association between miRNAs and the occurrence of immunological nonresponses, with the aim of searching for miRNA-based biomarkers for these HIV-1-infected individuals. Thirteen immunological responders (IRs) and 12 immunological nonresponders (INRs) were recruited, and RNA was collected from the plasma samples of the 25 HIV-1-infected individuals at both baseline and after 24 months of maintaining virological suppression (VS). Next-generation sequencing was used to detect miRNAs and evaluate the expression differences in miRNAs between IR and INR patients and between baseline and after 24 months of maintaining VS. Samples from 13 IRs and 11 INRs were successfully sequenced. The horizontal comparison of differentially expressed miRNAs between the groups and the longitudinal comparison of differentially expressed miRNAs between baseline and after 24 months of maintaining VS showed that a large proportion of miRNAs in INRs are downregulated compared to the levels in IRs. We also found that the miRNA let-7d-5p was downregulated in 9 INRs but only in 2 IRs by more than 2-fold. The difference was significant. In summary, these results demonstrate for the first time that a large proportion of miRNAs are downregulated in INRs compared with IRs, and the miRNA let-7d-5p is a potential biomarker for INRs.

## 1. Introduction

Acquired immune deficiency syndrome (AIDS) caused by human immunodeficiency virus (HIV) was recognized in the 1980s and has become one of the most devastating epidemics humanity has ever faced [1]. According to the Joint United Nations Programme on HIV/AIDS (UNAIDS), by 2018, there had been approximately 770,000 reported deaths due to AIDS-related illness among people living with HIV, and over 37.9 million people currently live with HIV [2]. However, despite a long battle against HIV, there is still no cure for this disease. Fortunately, due to the development of antiretroviral therapy (ART), AIDS, which was once terminal, has become a controllable chronic disease. In some developed countries, ART has extended the life expectancy of people living with HIV/AIDS to a length close to that of the HIV-negative population [3, 4].

However, despite the high efficiency of ART drugs, nearly 10-40% of HIV-1-infected patients suffer from the absence of CD4^+^ T cell count recovery despite sustaining full virological suppression (with undetectable HIV-1 viral load) after long-term ART [5]. These patients are considered immunological nonresponders (INRs) [6–9], indicating that their immune system did not respond to ART and reconstitute; prolonged immune depletion may lead to multiple AIDS- and non-AIDS-related clinical adverse events and present higher rates of mortality than HIV-1-infected individuals with adequate immune reconstitution [10].

The precise mechanism of INRs is still elusive. Current studies suggest that the risk factors for INRs are extremely broad, including decreased hematopoiesis of bone marrow, insufficient thymic output, residual viral replication, aberrant immune activation, delayed ART, and specific ART drugs [11, 12]. Because of the substantial complexity of the mechanisms underlying INRs, conventional research may not be the best method for solving one of the most pressing matters at hand, which is to find unified biomarkers to predict the occurrence of INRs. Thus, new insights have started to emerge due to the investigation of microRNAs (miRNAs).

miRNAs are small noncoding RNAs that guide a ribonucleoprotein complex to complementary RNAs to interfere with gene transcription [13]. miRNAs are involved in a large portion of the cellular activities in the human body and may serve as diagnostic identifiers and biomarkers with high levels of sensitivity and specificity [13]. Many studies have demonstrated the association between miRNAs and HIV pathology and immunity [14]. Certain miRNAs may even serve as diagnostic biomarkers [15–17], and some studies have proposed an association between the CD4^+^ T cell count of HIV-1-infected individuals and the alteration of their miRNA profiles [18, 19]. Meanwhile, with the improving awareness of HIV/AIDS among the general population, an increasing proportion of newly diagnosed patients are starting ART in the acute stage of HIV infection. The immunological and pathological profiles of patients in the acute infection stage are distinct from those in the chronic infection stage; however, most studies aimed at exploring the potential of miRNAs as indicators for CD4^+^ T cell counts were based on chronically infected patients. Here, for the first time, we studied the relationship between the miRNA profile and immune recovery of HIV-1-infected patients who started treatment in the acute infection stage.

## 2. Materials and Methods

### 2.1. Study Population

Twenty-five HIV-1-infected patients were enrolled in our study. Thirteen patients achieved complete immune reconstitution and were classified as the immunological responder (IR) group, while the other twelve patients were defined as experiencing immunological nonresponse and were classified as the INR group. The inclusion criteria for patients were as follows: (i) between the ages of 18 and 60 years; (ii) recruited from the Beijing PRIMO clinical cohort, with HIV-1-infected men who have sex with men (MSM); (iii) initiated ART in the acute HIV infection stage (defined as positive HIV RNA with negative HIV antibody [20]); (iv) received ART at Beijing Youan Hospital for 24 months or longer; (v) achieved virological suppression (VS) within 12 weeks after initiating ART and maintained VS for at least 24 months; (vi) received the first-line ART regimen according to China's National Free Antiretroviral Treatment Program (NFATP) and the Chinese Center for Disease Control and Prevention guidelines [21]; and (vii) had no coinfection with HBV or HCV and had no occurrence of serious AIDS and non-AIDS-related clinical adverse events.

Currently, there is no worldwide consensus on the definition of incomplete immune reconstitution. In our study, we defined INRs as HIV-1-infected patients with CD4^+^ T cell counts ≤ 350 cells/*μ*l and IRs as those with ≥500 cells/*μ*l after at least 24 months of ART with VS [22]. All patients received the combination of lamivudine, efavirenz, and tenofovir disoproxil fumarate as the regimen.

This study and all the relevant experiments were approved by the Beijing Youan Hospital Research Ethics Committee (2018-035), and written informed consent was obtained from each participant in accordance with the Declaration of Helsinki. All participants provided written informed consent for the collection of information, and their clinical samples were stored and used for research. The methods used conformed to the approved guidelines and regulations.

### 2.2. Nucleic Acid Extraction, Library Construction, and Sequencing

Fifty plasma samples (with EDTA anticoagulant) were collected from all 25 patients (13 in the IR group and 12 in the INR group) at both baseline and after 24 months of maintaining VS and frozen. Two hundred microliters of plasma from each sample was used for this study. Nucleic acid extraction was performed using the miRNeasy® Mini Kit (Qiagen, Catalog number 217004) following the manufacturer's instructions. Extracted nucleic acids were RNA fragments < 200 nucleotides long, including miRNA. The elution volume of enriched RNA fragments was 20 *μ*l with RNase-free water for each sample, and the samples were stored at -80°C before library construction.

The small RNA sequencing process was based on the Illumina® BGISEQ500 platform and performed by Beijing Mygenostic Co., Ltd. We used an Agilent 2100 Bioanalyzer (Agilent Technologies, USA) to detect and estimate the quality (RIN ≥ 7) and concentration of the small RNAs acquired from the last step. Then, we evenly divided each sample into three portions. Equal amounts of total small RNA diluent from each portion from both groups and both time points were then pooled for library construction. All portions were pooled at the same time to minimize bias.

Then, library construction was performed according to the following steps: (1) polypropylene acyl amine gel electrophoresis (PAGE) was used to enrich and purify the small RNAs with lengths below 18 nt or above 30 nt; (2) the 3' ends of the purified small RNAs were attached to 5-adenylated, 3-blocked single-stranded DNA adapters; (3) the reverse primer and 5' adaptors were hybridized to the 3' and 5' ends of the small RNAs, respectively; (4) cDNA was synthesized by Superscript II reverse transcriptase (Invitrogen, USA); (5) the cDNA was enriched using both 3' and 5' adaptors; (6) the cDNA library was purified using PAGE; and (7) an Agilent 2100 Bioanalyzer (Agilent Technologies, USA) was used for quantification of the library. Finally, the prepared cDNA libraries were sequenced on the Illumina® BGISEQ500 platform.

### 2.3. microRNA Identification and Analysis

After the initial sequencing, the data were first purified to exclude the most low-quality, invalid adapters, short valid tags, and all PolyA tags. After the process, we acquired sufficient valid reads for small noncoding RNA identification and quantification.

All purified sequencing results (containing known miRNAs, novel miRNAs, genomic miRNAs, and other small noncoding RNAs and RNA fragments from unknown sources) were compared with several databases, including miRBase (http://www.mirbase.org/), Rfam (http://rfam.xfam.org), siRNA (http://web.mit.edu/sirna/), piRBase (http://www.regulatoryrna.org/), and snOPY (http://snoopy.med.miyazaki-u.ac.jp/), to identify miRNAs and their target genes. The novel miRNAs were defined by searching the 5 databases mentioned above, allowing a maximum of 2 mismatches, while known miRNAs were exactly conserved.

The detected miRNAs were quantified for analysis. The expression differences (upregulation and downregulation) of miRNAs were compared for each patient between baseline and after 24 months. The results are displayed as log_2_ fold changes. All data with false discovery rates above 0.001 were excluded.

We compared the miRNA profiles between baseline and 24 months after achieving VS for each patient and selected miRNAs that were upregulated or downregulated by more than 2-fold (log_2_*X* > 1 or <-1). Then, we compared the altered miRNAs between the IR and INR groups.

### 2.4. Statistical Analysis

All statistics analyses and calculations were performed using Microsoft Office Excel 2013 and GraphPad Prism software v5.03 (GraphPad Software, San Diego, CA, USA). In this article, statistical significance was defined as a *p* value <0.05 using *t*-tests, Mann–Whitney *U* tests, or Fisher's exact tests as appropriate. All *p* values are two-tailed.

## 3. Results

### 3.1. Patient Characteristics

Twenty-five patients were enrolled as study subjects. The library construction failed on one baseline sample of those patients due to insufficient quality, and that patient was then excluded from our study. Among the remaining 24 patients, 13 patients were classified in the IR group, while 11 patients were classified in the INR group. There was no significant difference between the two groups at baseline in terms of age, viral load, and CD4^+^ T cell counts. The characteristics of the patients are presented in Table 1.

### 3.2. Sequencing Data Analysis and microRNA Identification

**Figure 1:**
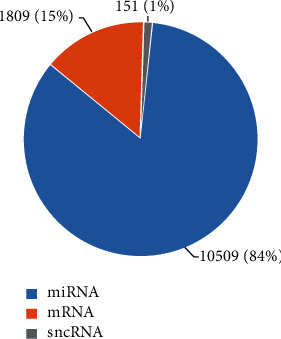
Mapped small RNA sequences. The numbers presented are the average of a thousand tags in all samples. miRNA: microRNA; mRNA: messenger RNA; sncRNA: (other) small noncoding RNA.

After sequencing, the sequencing data were first purified to exclude the majority of low-quality, invalid adapters, short valid tags, and all PolyA tags and yield a mean of 24,144,826 ± 4,993,513 clean tags (each read had one tag attached to it; thus, the tag number equals the read number) in all samples showed by the SE50 sequence type. At least 98.3% and up to 99.5% of the final clean tags in each sample had a quality score above 20, which was sufficient for small noncoding RNA identification and quantification. After data purification, we compared the clean data with several RNA databases mentioned earlier to map the purified tags. The percentage of mapped clean tags in each sample was above 70%, except for one sample collected from a patient in the IR group after 24 months of VS, which mapped only 42.35%. Overall, the mapped sequences were categorized into small pieces of genomic mRNA (1809, 15%), including introns (5%), exons (4%), repeats (2%), intergenic mRNAs (3%), and miRNA (84%), including hairpin structures (46%), precursors (4%), mature miRNAs (34%), and other small noncoding RNAs (1%, siRNA, piRNA, etc.) (Figure 1). The percentages presented above are the average proportions of certain small RNAs among all mapped tags in all samples.

In total, we identified 1341 known miRNAs in the databases and predicted 5281 potential novel miRNAs that were regulated between the INR and INR groups or between baseline and after 24 months of VS.

### 3.3. Different microRNA Profiles between the Groups

**Figure 2:**
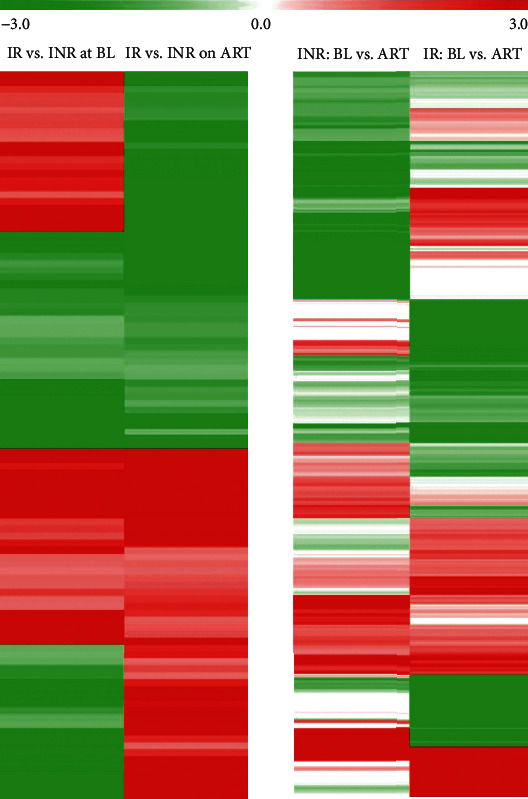
Hierarchical clusters of differentially expressed known microRNAs between the IR and INR groups at baseline and after 24 months of ART. BL: baseline; ART: after 24 months of ART.

To investigate the differences in the miRNA expression profiles between the groups, we pooled all samples of each group and performed a hierarchical analysis of miRNA expression to conduct horizontal comparisons between the INR and IR groups. The differentially expressed known miRNAs at baseline and after 24 months of VS were analyzed separately. Overall, 620 miRNAs were differentially expressed between the groups (299 at baseline and 321 after 24 months of VS, as presented in the left panel of Figure 2). However, if we consider only the miRNAs that showed the same regulation pattern (up- or downregulation at both baseline and after 24 months of VS in the INR group compared to the IR group), the number of regulated miRNAs decreased to only 120.

**Figure 3:**
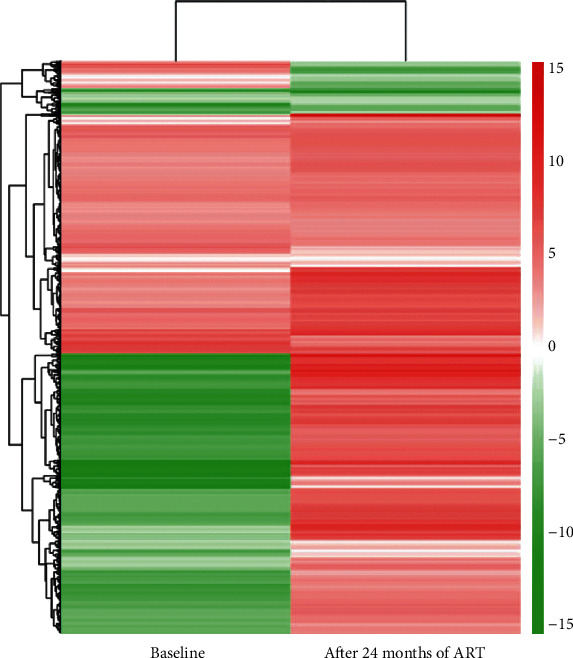
Hierarchical clusters of all differentially expressed microRNAs between the IR and INR groups at baseline and after 24 months of ART.

Although there is no clear regulation pattern of known miRNAs in the INR and IR groups, after including the novel miRNAs detected in the study, we found that among 2,009 miRNAs that had lower expression in the IR group compared to the INR group at baseline, 1,230 of them were upregulated by greater amounts and exceeded the expression level in the INR group (Figure 3).

### 3.4. Regulation of microRNAs between Baseline and after 24 Months of Virological Suppression

To further evaluate the different miRNA profiles between the IR and INR groups, we conducted a longitudinal analysis to investigate the different regulation patterns of miRNAs between IR and INR patients. We analyzed the differentially regulated miRNAs in the two groups. In total, 1200 miRNAs were up- or downregulated after 24 months of VS compared to the baseline values (959 and 366 in the IR group and INR group, respectively, as presented in the right panel of Figure 2). The pooled analysis did not show a clear pattern that could suggest an association between miRNA regulation and the occurrence of INR.

However, to investigate the potential of miRNAs to be used as biomarkers for INR and indicators for CD4^+^ T cell count, we analyzed the miRNA regulation profiles in each patient to evaluate whether there are different regulation patterns in the IR and INR groups. The results showed that a certain miRNA, let-7d-5p, was downregulated in 9 of the 11 INRs by more than 2-fold (one upregulated and 1 unchanged), while it was downregulated by more than 2-fold in only 2 of the 13 IRs (2 upregulated, 9 unchanged or downregulated less than 2-fold, *p* = 0.0031). A similar pattern was not found for the other miRNAs.

### 3.5. Target Genes of Differentially Expressed miRNAs

**Figure 4:**
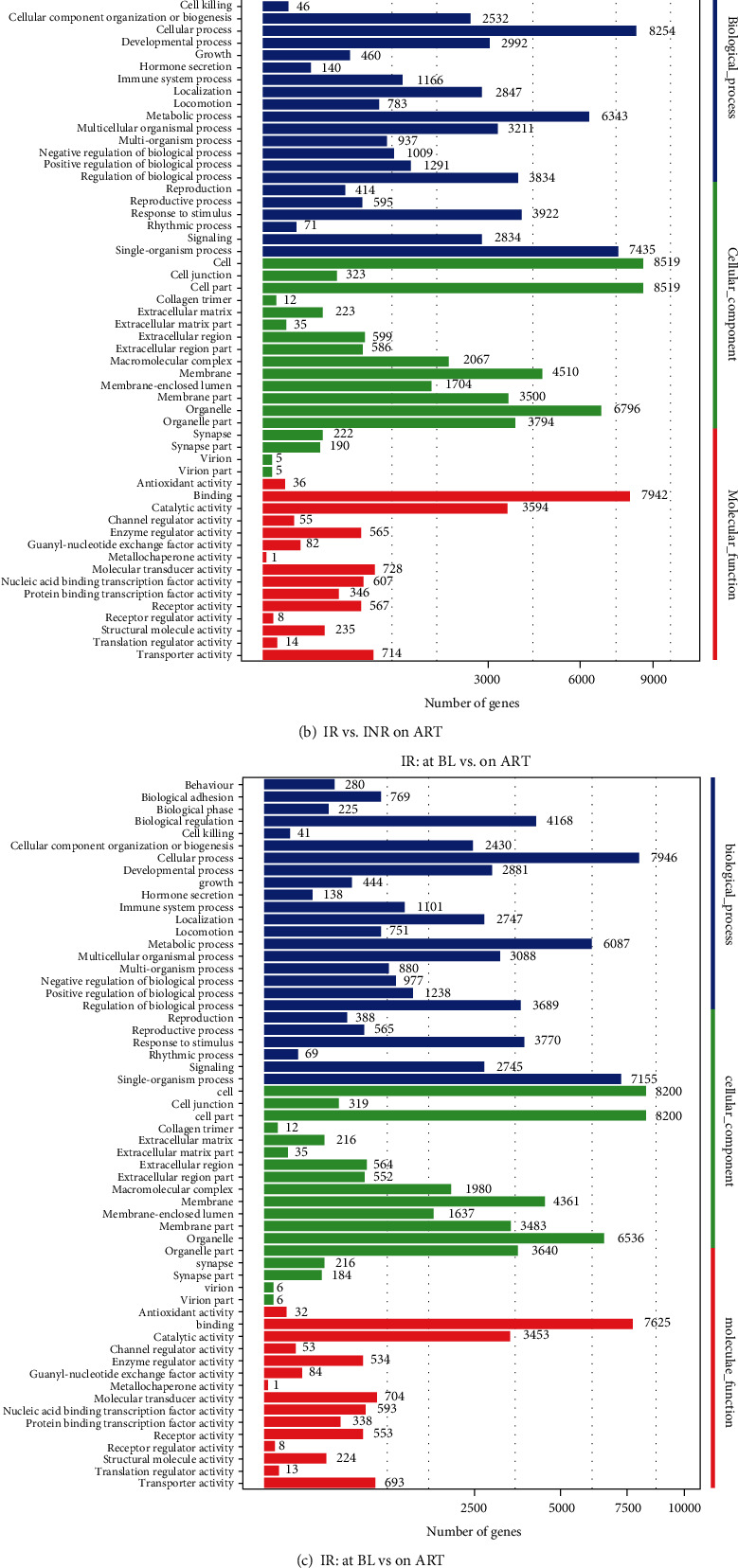
Target genes of differentially expressed miRNAs between baseline and after 24 months of ART. The target genes of the differentially expressed miRNAs between the IR and INR groups at baseline (a), the IR and INR groups after 24 months of maintaining VS (b), at baseline and after 24 months of maintaining VS in the IR group (c), and at baseline and after 24 months of maintaining VS in the INR group (d). Blue columns indicate the target genes involved in biological processes; green columns indicate the target genes associated with cellular components; red columns indicate the target genes involved in cellular activities/functions. VS: virological suppression; IR: immunological responders; INR: immunological nonresponders; BL: baseline; ART: antiretroviral therapy (at least 24 months of ART).

To further evaluate the impact of differentially expressed miRNAs, we compared the target human genes of the identified miRNAs in this study through the three miRNA databases mentioned previously. The targeted genes were classified into three groups: genes involved in biological processes, genes involved in cellular activities/functions, and genes regulating cellular components. Then, the target genes were horizontally and longitudinally compared between and within the IR and INR groups at baseline and after over 24 months of ART. The results are shown as the number of target genes by gene function. The most affected genes are those involved in single-organism processes, cellular processes, cellular structure, organelle structure, and cell binding. The nonpaired comparisons of the pooled analysis of four whole groups (IRs at baseline, IRs after 24 months of ART, INRs at baseline, and INRs after 24 months of ART) are presented in Figure 4.

## 4. Discussion

Because HIV infection is increasingly considered a chronic disease rather than a fatal one and a cure for HIV infection is still a long way from realization, improving the quality of life of HIV-infected patients has become a key issue. Thus, the center of that issue is to reconstruct a fully functional immune system in patients.

INRs have drawn increasing attention from researchers and first-line physicians ever since this concept was first postulated in 2003 [23]. Recent studies have shown that the risk factors for immune nonresponse widely vary [5]. Most studies on INRs have focused on isolated factors, and none of those are convincing when used to explain the occurrence of INRs. Meanwhile, as limited as our understanding of INR, we still need a simpler and more direct tool to assess INR for clinical purposes, and miRNAs might precisely provide that tool.

The results of the horizontal hierarchical analysis of known miRNAs in our current study showed little correlation between INR and miRNA regulation. However, if we included the novel miRNAs, a considerable proportion of miRNAs had lower expression levels in IRs than in INRs at baseline (lower left clusters in Figure 3). However, after over 24 months of ART and maintaining VS, the expression levels of the majority of those miRNAs in the IR group exceeded those in the INR group (lower right clusters in Figure 3). By combining the horizontal and longitudinal results, it is reasonable to assume that a large number of miRNAs are more strongly downregulated in INRs than in IRs. According to previous studies, the miRNAs in peripheral blood mononuclear cells (PBMCs) and serum are universally downregulated due to HIV infection [24, 25], while the majority of differentially expressed miRNAs in our study were downregulated, especially in the INR group. This indicates that the former studies partially supported our results, although the patterns of miRNA regulation in serum could be different from those in PBMCs, as miRNAs in serum are generated from a broader range of sources. Unfortunately, there are not enough published studies on the association between INRs and miRNAs let alone in patients who started ART in the acute stage of HIV infection to confirm these results. Moving forward, we should cover more follow-up time points to accomplish better surveillance of miRNA expression in INR patients and compare results between serum and PBMCs in future studies. Single-cell sequencing could also be a promising approach to investigate the involvement of miRNAs in immune reconstitution. In addition, since we have identified a great number of novel miRNAs, it is easier to target those miRNAs in future studies and maybe even confirm the association between INR and those miRNAs.

Meanwhile, our individual analysis showed that the miRNA let-7d-5p, the most highly differentially expressed miRNA between the groups, was downregulated in the INR group but not in the IR group. This miRNA belongs to the let-7 family, which was the first miRNA family discovered, as it was identified even prior to the conception and naming of miRNAs as an entity. This heterochronic regulatory RNA family is also one of the most well-studied miRNA families and is involved in a wide range of human physiological activities, especially cell development. Previous studies demonstrated that the inhibition of the let-7 family may cause progenitor cell proliferation and interfere with erythroid development [26, 27]. Erythroid cells are known to promote the development of regulatory T cells, which are correlated with CD4^+^ T cell recovery [27–29]. This may explain the association between the downregulated let-7d-5p and INRs.

However, when we pooled the samples and performed comparisons as groups, the miRNA let-7d-5p did not show differential expression between the groups in the longitudinal or horizontal comparisons, although individual miRNA expression differences being neutralized during sample pooling are not entirely unexpected.

We should also note that in the current study, thousands of miRNAs were identified, which means that although certain miRNA expression levels could be significantly different between groups, it could still be biased due to the large sample size. There is a chance that similar differences can be found in any randomly generated data with a similar volume or even a lower volume. Therefore, we should expect to confirm this finding in further studies instead of jumping to conclusions.

For the gene-level analysis, we identified the target genes of the differentially expressed miRNAs in this study. Interestingly, after horizontal and longitudinal comparison, we found that all the comparison results were almost identical, and the affected genes covered almost the whole range of miRNA regulation profiles. Thus, we were unable to ascertain the exact impact of miRNA regulation at the phenotypic level. However, this result suggested that despite ART and immune reconstitution, the miRNA profile is constantly altered from time to time and from patient to patient, which raises a new challenge for us, which is to develop a better system for the surveillance of miRNAs in patients, as most current miRNA sequencing and identification techniques are developed based on DNA sequencing methods and lack phenotypic study. A well-designed cohort for INRs should also be very beneficial.

In this study, we chose to use next-generation sequencing (NGS) as a miRNA detection method instead of the more commonly used microarray. Although microarrays have been shown to possess higher sensitivity than NGS for detecting miRNA [30], they can only detect known miRNAs with a designed probe. Moreover, it is almost impossible to design a chip with a detection range and capacity to cover all the miRNAs that we detected with NGS. More importantly, the microarray method cannot detect different miRNA variants, while NGS can [31, 32]. The association between INR and miRNAs has rarely been studied; given the explorative nature of the current study, we would like to obtain a larger picture of the whole miRNA profile of INRs, as well as the sequences of novel miRNAs, for future full-scale studies.

Our study also has some limitations. First, there was no healthy control group in our study for cost-efficiency reasons, and the miRNA data of healthy individuals currently published online was not satisfactory for the current study. Additionally, the sequencing method we chose could not provide the exact expression values of certain detected miRNAs, and all analyses were based on expression changes. However, the current results did narrow down the candidates for miRNAs that are associated with poor immune reconstitution in HIV-1-infected patients and provide vital information for us to use when designing suitable chips or using other methods to further investigate the INR-associated miRNAs that drew our interest. Additionally, we did not perform transcriptional analysis for verification due to the lack of required samples.

## 5. Conclusion

In summary, we found that a large proportion of miRNAs are downregulated in INRs compared with IRs after 24 months of ART. In addition, we found that the miRNA let-7d-5p is downregulated in INRs but not in IRs in the current study, and it could be a potential biomarker for INRs after full-scale validation.

## Figures and Tables

**Table 1:** Characteristics of the participants enrolled in this study.

Characteristics	INRs (11)	IRs (13)	Total (24)	*p* value
Patient age (years) at BL	29.0 ± 7.4	33.0 ± 7.4	31.2 ± 7.5	0.1293
VL, log_10_ copies/ml at BL	4.5 ± 0.6	4.6 ± 0.7	4.5 ± 0.6	0.8646
CD4 (cells/*μ*l) at BL	286.8 ± 59.7	278.2 ± 95.8	282.2 ± 79.7	0.4585
CD4 (cells/*μ*l) after 24 month ART	304.1 ± 33.0	786.2 ± 193.2	565.3 ± 283.1	<0.0001

BL: baseline; VL: HIV viral load; SD: mean ± standard deviation.

## Data Availability

The datasets during and/or analyzed during the current study are available from the corresponding authors on reasonable request.

## Abbreviations

HIV: Human immunodeficiency virus
AIDS: Acquired immune deficiency syndrome
ART: Antiretroviral therapy
miRNA: microRNA
IRs: Immunological responders
INRs: Immunological nonresponders
VS: Virological suppression
MSM: Men who have sex with men
NFATP: China's National Free Antiretroviral Treatment Program
PAGE: Polypropylene acyl amine gel electrophoresis.
